# The Relationship between Bodyweight Status and Weight Perception Explains Differences in Calories Ordered in a Food Choice Exercise

**DOI:** 10.3390/nu13061794

**Published:** 2021-05-25

**Authors:** Jean-Claude Mbarushimana, Christopher R. Gustafson, Henriette Gitungwa, Eliana Zeballos

**Affiliations:** 1 College of Agricultural Sciences and Natural Resources, University of Nebraska-Lincoln, Lincoln, NE 68583, USA; jean-claude@huskers.unl.edu; 2 Department of Agricultural Economics, University of Nebraska-Lincoln, Lincoln, NE 68583, USA; hgitungwa@huskers.unl.edu; 3 USDA Economic Research Service, Washington, DC 20250, USA; eliana.zeballos@gmail.com

**Keywords:** food choice, calories, BMI, perceived weight status, relative weight status, perception

## Abstract

Understanding food choice is critical to be able to address the rise in obesity rates around the globe. In this paper, we examine the relationship between measured (BMI, using self-reported height and weight) and perceived weight status with the number of calories ordered in a controlled online food choice exercise. A total of 1044 participants completed an online food choice exercise in which they selected ingredients for a sandwich from five categories: meat/protein, cheese, spread/dressing, bread, and vegetables. We examine the number of calories ordered by participants and use linear regression to study the relationship of BMI category relative to self-reported perceived weight status with calories ordered. As a comparison to previous literature, we also examine the relationship between relative weight status and self-reported dieting behavior using logistic regression. We find that participants perceiving themselves to have a higher BMI than their BMI calculated using height and weight ordered significantly fewer calories and were more likely to report dieting than participants who perceived themselves to have a lower BMI than their calculated BMI. The relationship between perceived weight status and measured weight status explains behavior in a food choice task. Understanding how people perceive their weight may help design effective health messages.

## 1. Introduction

The prevalence of overweight and obesity among American adults has increased markedly over the past few decades, from 56.0 percent in 1988–1994 to 73.1 percent in 2017–2018 [1]. Increases in overweight and obesity are a threat to public health as both overweight and obesity have been positively associated with chronic diseases, lower quality of life, decreased life expectancy, and higher health care costs [2,3,4,5].

Dieting (limiting or changing the intake of food to lose or maintain weight) and physical activity are frequently promoted as ways to lose weight or prevent additional weight gain. These attempts are frequently unsuccessful, which may be due to a lack of adherence to the weight loss program undertaken [6,7]. However, it is not yet clear what motivates individuals to follow planned weight loss activities. Researchers have not found consistent predictors of dropouts from these programs [8], and are still establishing what prompts people to undertake weight-loss activities. Findings suggest several factors such as gender, individual perception of ideal weight, experiences of discrimination, weight goals, and social support, among others [9,10,11,12,13].

A motivator that has been documented in initiating weight loss programs is an individual’s perception of their weight status [13,14,15,16]. BMI is typically used to measure and categorize individuals’ weight status by healthcare professionals and researchers. This information, however, is not necessarily communicated to the patient. In a study using data from the National Health and Nutrition Examination Survey (NHANES), three-quarters of overweight and nearly one-third of obese individuals reported that they had never received a diagnosis of overweight/obesity [16]. While healthcare-based nutrition counseling is based on measured weight status, research suggests that physicians face barriers to providing nutrition counseling [17], so individuals’ perceptions of their weight status—rather than their weight status based on their BMI—may more frequently influence individuals’ decisions to undertake weight loss or weight maintenance activities.

Misperceptions about weight status may also be important in understanding weight loss/maintenance strategies. First, there is evidence that BMI calculated from self-reported height and weight is lower than BMI from measured values [18]. People tend to under-report their weight and over-report their height [19]. This results in discrepancies between objectively measured and self-reported BMI. Second, people may have perceptions of their weight status—whether they are underweight, normal weight, overweight, or obese—that differ from their calculated BMI, even if they know their objective height and weight measures. BMI is calculated using a formula that is complex enough that individuals may not be able to simply carry out the calculation in their heads and may not take the time to calculate their BMI from their height and weight. A study of internal medicine residents—highly trained physicians whose work will likely require them to treat obese patients on a regular basis—showed that over one-third of these residents incorrectly calculated their own BMI by more than 10% (even with the use of a calculator) [20]. More than half of the residents did not know the BMI cut-off value that leads an individual to be classified as obese [20]. Other studies have found similar discrepancies between individuals’ perceived weight status and BMI measures obtained using either self-reported or objectively measured height and weight data among samples drawn from the general public [16,21].

Studies have documented discrepancies between individuals’ perceived weight status and objectively reported weight status, finding that a significant number of overweight and underweight people perceive themselves to be normal weight [16,21]. For instance, a study conducted with high school students found that a significant number of overweight students perceived themselves to have a lower BMI than what was obtained using either self-reported or objectively measured height and weight values [21]. These discrepancies present further public health risks since individuals’ weight status perceptions influence their weight management decisions. Multiple studies have found that individuals’ weight perceptions are related to habitual weight management attempts, such as dieting or exercise [14,15,16]. This means that overweight individuals who perceive themselves to be normal weight may be less likely to undertake a weight loss/maintenance program.

Perceived weight status may be an important driver of people’s attempts to control their weight through food choices. While the studies mentioned above have documented a relationship between perceived weight status and self-reported habitual behaviors, such as exercising or dieting, we have been unable to find any studies examining the relationship between perceptions of weight status and direct measures of behavior, such as individual food choice, in a controlled choice setting. In this study, we address this gap in the literature by examining the relationship between differences between perceived body weight status and body weight status calculated from self-reported weight and height and the number of calories ordered in a food choice task. To provide a comparison of our findings with previously published analyses examining self-reported health behaviors—such as dieting and exercise—we additionally analyze data on whether participants report that they are dieting using measured and relative body weight status. This analysis provides a novel examination of food choice behavior by people with different perceptions of their weight status in a controlled environment, while corroborating previous studies’ findings that perceived weight status is important in explaining self-reported health behaviors.

## 2. Materials and Methods

### 2.1. Data Collection

This study uses data from an online experiment that was conducted with participants recruited from Amazon’s Mechanical Turk (mTurk) between 24 April and 3 May 2018. Participants completed a hypothetical sandwich choice experiment and then responded to a survey, which included questions about demographic information, health, and nutrition behaviors [22,23]. Respondents were required to be United States residents of at least 19 years of age and were only allowed to complete the survey once. Participants received $3.00 for completing the survey. The Institutional Review Board of the University of Nebraska-Lincoln approved the research (IRB protocol #20171017580EX). All participants provided written informed consent before participating in the research.

### 2.2. Survey Design

In the experiment, participants selected the ingredients for a sandwich, which they were instructed to imagine they were subsequently going to eat, from five categories: (1) meat/protein, (2) cheese, (3) spread/dressing, (4) bread, and (5) vegetables. The calorie information was provided for the amount of each ingredient included in the sandwich. For instance, if an individual chose to include bacon in the sandwich, that choice would add 254 calories to the sandwich (see Table 1). Respondents also were given access to information about the amount of each item that would be included in the sandwich (e.g., four ounces of ham). Participants were allowed to select one item from each category, which is common practice at build-your-own sandwich restaurants. The types and quantities of ingredients included in the sandwich choice task were based on sandwich ingredients and serving sizes from a sandwich shop with locations throughout the US that provided data for a natural experiment on per-ingredient versus per sandwich calories labeling [24]. We used the United States Department of Agriculture Food Composition Database to retrieve calorie information for each ingredient.

The ingredients that participants could select from—and the number of calories that each item would add to the sandwich—are presented in Table 1. Participants all saw the categories of ingredients displayed in the same order, which corresponds to the order in which they were displayed at a sandwich counter studied in previous research [24]. Participants were not required to select an ingredient from each category; they had the option to indicate that they would not select an ingredient in a particular category if they did not want to add any of the available ingredients. We obtained calorie information for ingredients from the United States Department of Agriculture Food Composition Database [25] based on the amount (e.g., ounces) of the ingredient added to sandwiches at the sandwich counter of a national food retailer chain [24]. The survey randomly assigned participants to one calorie information condition. The various conditions are described in Appendix A. We checked for successful randomization of individuals to condition by testing for differences in individual characteristics—including BMI—across conditions using Bonferroni adjustments for multiple comparisons. The key outcome variable in the research is the total number of calories in the sandwich that participants build in the sandwich choice exercise.

Finally, participants completed a section of the survey containing demographic, health, and nutritional questions. Two key independent variables in our analysis are weight status based on BMI calculated from self-reported height and weight data and perceived weight status, in which participants were asked if they considered themselves to be underweight, normal weight, overweight, or obese (or if they did not know). An individual’s perceived weight category was compared to the category obtained from calculating BMI based on the participant’s self-reported height and weight to identify differences between calculated (based on self-reported weight and height measures) and perceived weight status. Importantly, height and weight measures and the perceived weight question were collected back-to-back in order to minimize discrepancies between measured and perceived height and weight status. First, participants were asked to enter their height without shoes on and then their weight without shoes. The researchers used the self-reported weight and height to calculate the individual’s BMI and then classified individuals into BMI categories: underweight = BMI < 18.5; normal weight = 18.5 ≤ BMI < 25; overweight = 25 ≤ BMI < 30; and obese = BMI ≥ 30. Finally, participants reported whether they considered themselves to be underweight, normal weight, overweight, or obese.

### 2.3. Data and Statistical Analyses

We examined the relative relationship between measured and perceived weight status in in the analyses. In order to directly capture the relative relationship between measured and perceived weight status, we created a new variable that describes the relative relationship between the calculated BMI category and the perceived BMI category. The relative variable captures the relationship of the individual’s perception of their weight status to the calculated weight status—that is, if their perception matches their weight status by measurement (*Accurate*), if they think they fall into a lower weight category than they do by measurement (*Underestimate*), or if they believe they fall into a higher weight category than their measurement-based category (*Overestimate*).

We analyzed the data using summary statistics and ordinal and linear regression analyses. We reported summary statistics for demographic characteristics of the participants, and then examined the relationship between measured and perceived weight status. Next, we conducted a logistic regression of reported dieting by participants’ measured and relative weight status. We include this analysis for comparison with previous literature examining the relationship between perceived body weight and habitual weight-management activities. Next, we used linear regression to analyze the number of calories ordered in the food choice exercise by measured and relative weight status. In the analysis of calories selected, we included control variables for the information conditions. Because the relative measures can only take all of the values (under, accurate, or over-estimate of measured weight status) for normal weight or overweight individuals, we additionally performed these analyses on a subset of the data that includes individuals who are normal weight or overweight by calculated BMI. For analyses on the full dataset, we combined the measured weight category of underweight, which constituted only 26 participants, with the normal weight category. Data analysis was conducted in R [26].

## 3. Results

### 3.1. Summary Statistics

Table 2 represents summary statistics on the study sample. The number of respondents to the survey was 1044. Just over half—51%—of the participants were female. The average age of participants was approximately 37 years, and average education was slightly less than 15 years, which is approximately equivalent to an Associate Degree. The average household pre-tax income was slightly over $52,000. Values are quite similar for the subset of individuals who were normal weight or overweight. There were no significant differences in individual characteristics among the calorie information conditions.

### 3.2. Calculated Weight Status versus Perceived Weight Status

Based on calculated BMI—using self-reported data on participants’ height and weight, 2.5 percent of participants were underweight (BMI < 18.5), 40.9 percent were normal weight (18.5 ≤ BMI < 25), 28.6 percent were overweight (25 ≤ BMI < 30), and 27.9 percent of participants were obese (BMI ≥ 30). Next, we examined participants’ perceptions of their weight status. A little over three percent of participants reported that they perceive themselves to be underweight, 44.3 percent reported that they perceive themselves to be normal weight, 39.0 percent reported perceiving themselves to be overweight, and only 13.5 percent reported a perception of being obese. These results demonstrate that BMI based on self-reported height and weight information and self-reported perceived BMI status do not perfectly align.

Table 3 compares calculated weight status to perceived weight status. Next, we examine the relationship between calculated and perceived weight status. Nearly sixty percent (57.7%) of underweight participants accurately perceived themselves to be underweight, while 42.3 percent of them over-estimated their weight status, perceiving themselves to be normal weight. Over four-fifths (82.8%) of normal weight participants accurately perceived their weight status, while 12.9 percent overestimated their weight status (i.e., perceived themselves to be overweight) and 4.3 percent underestimated their weight status (i.e., perceived themselves to be underweight). Among overweight participants, 69.2 percent accurately perceived themselves to be overweight, while 29.1 percent underestimated their weight status and 1.7 percent overestimated their weight status. For obese participants, 50.2 percent accurately perceived their weight status, while nearly half—49.8 percent—underestimated their weight status. Among obese participants who underestimated their weight status, 93.5 percent said they were overweight while 6.5 percent thought they were normal weight.

### 3.3. Relationship between Weight Status and Dieting

Table 4 reports the relationship between weight status and dieting from a binary logistic regression for the full sample and for the subsample of participants who were normal weight or overweight by measurement. In the analysis of the full sample, participants who were obese based on calculated BMI weight status were 3.4 times more likely to diet than those who were normal weight (which is the omitted weight category). Likewise, participants who were overweight were 2.6 times more likely to diet than those who were normal weight. When we examine the relationship that the discrepancy between perceived and calculated BMI status has with dieting behavior, we find that participants who accurately perceived their weight status were 1.7 times more likely to diet than participants who underestimated their weight status. Those who overestimated their weight status were 2.7 times more likely to report dieting compared to participants who underestimated their weight status.

In the analysis of the sub-sample of normal and overweight participants, we see the same relative relationships, though with slightly moderated estimates. Individuals who were overweight were slightly under 2.5 times more likely to report dieting than individuals who were normal weight. Participants who accurately estimated were 1.5 times more likely to report dieting than those who underestimated their weight, while those who overestimated their weight status were 2.35 times more likely to report dieting.

### 3.4. Number of Calories Ordered in a Food Choice Exercise

Table 5 reports the relationship between weight status and calories ordered, first in the full sample and then in the subsample of normal and overweight individuals. In each case, we include indicator variables to control for the information conditions. Measured weight status is not significant in any of the analyses. However, discrepancies between perceived weight status and measured weight status on calories ordered are significant. In the full sample, those who overestimated their weight status ordered 46.7 calories fewer than individuals who underestimated their weight status, which is statistically significant at *p* < 0.02. In the subsample of normal and overweight individuals, both individuals who accurately perceived their weight status (32 fewer calories, *p* = 0.025) and those who overestimated their weight status (81 fewer calories, *p* < 0.001) ordered significantly fewer calories than individuals who underestimated their weight status.

We do not have data about the state of hunger of respondents; we do, however, have data on how long it had been since they had last eaten. We have included that variable in the analyses we conducted, but, while positive, the estimated coefficient is very small, is not statistically significant, and does not affect the estimates of the variables of interest. These estimates are provided in Appendix B Table A1.

## 4. Discussion

In this article, we compare calculated and perceived weight status to participants’ reports of being on a diet and to the total number of calories ordered in the sandwich choice experiment. The novel contribution of our study is that we find evidence of differences in observed behavior in the food choice task. Participants who overestimated their weight status ordered significantly fewer calories than individuals who underestimated their weight status—a reduction amounting to nearly 13 percent of the mean number of calories ordered per sandwich in the research in the subsample of normal and overweight individuals. These findings may also explain why we do not observe significant differences in calories ordered by calculated BMI category. Previous research has documented that individual variation in motivations explain differences in information-seeking behavior and food choice [27,28]. In our results, perceived weight status appears to add a key piece of information for understanding differences in food choices. We also find strong evidence that individuals who overestimated their weight status were significantly more likely to be on a diet.

Our study corroborates the findings of previous studies that examined habitual weight control activities, such as dieting and exercise. Prior studies have examined the relationship between weight status perception and individuals’ decisions regarding attempts at weight loss or maintenance. Overweight and obese adults who did not perceive themselves to be overweight were less likely to want to weigh less and were less likely to attempt weight loss activities compared to those who perceived themselves to be overweight [29]. In another study, accurate perceivers were more likely than those who mis-perceived their weight status to report weight loss intention [30]. Perceiving oneself to be overweight influences individuals to attempt weight management programs [14]. We find similar results. We examine the influence of weight status on dieting, and participants who were overweight and obese by measurement were more likely to diet than participants with normal weight, and those who accurately or overestimated their weigh status were more likely to diet than those who underestimated it, even while controlling for weight status.

Our study adds to previous studies by examining behavior in a specific instance of food choice. We examine the number of calories individuals who overestimated their weight statuses ordered compared to those who underestimated them. Individuals who accurately estimated and overestimated their weight status ordered around five percent and 13 percent fewer calories, respectively, than individuals who underestimated their weight status when we examine normal and overweight participants.

Weight overestimation has been attributed to different sources. Only a narrow range of body shapes and sizes are accepted in western culture [31]. Perceived pressure to be thin increases risks for body dissatisfaction and leads to an increase in dieting behavior to retain the ideal shape. In fact, while we find that accurate and overestimation of one’s body size is associated with fewer calories being ordered in a food choice exercise, overestimating weight perception can be unhealthy, putting individuals at risk of developing eating disorders. Individuals may place themselves on an unnecessarily restrictive diet, hence limiting the intake of critical nutrients needed for healthy functioning of the body [32]. Multiple studies have found that overestimating one’s weight status was positively correlated with eating disorders [33,34,35,36].

For health and weight management recommendations such as dieting or limiting calorie intake—or identifying individuals at risk of developing eating disorders—it may be important to screen individuals’ perceived weight status, in addition to calculating their BMI, when providing behavioral recommendations. Our research highlights the importance of perception in undertaking health behaviors. An upshot is that the effectiveness of public health messages about weight maintenance or weight loss activities may depend on individuals’ perceptions as much as—if not more than—it does on the objective measures typically relied upon by health professionals and researchers [37]. Efforts to influence individuals’ food choice behaviors to avoid health complications associated with overweight and obesity may want to consider perceptions of weight statuses. Otherwise, public health campaigns may be ineffective.

Our study—and other papers on the effect of perceived body weight on health behaviors—contributes to a broader literature on the role of subjective beliefs in healthy food choice. Recent work examining factors that influence individuals who do not have a diagnosed reason to follow a gluten-free diet shows that positive beliefs about the health benefits of gluten-free (vs. conventional) foods are highly related to following the diet [38]. Other studies have documented a tendency among fast-food consumers to underestimate the content of nutrients that should be limited, such as calories [39] and sodium [40], in the meals they have consumed. Recent work suggests that these incorrect beliefs do affect behavior, but correcting inaccurate beliefs about nutrients can lead to healthier subsequent choices [23,41].

While we find consistently significant effects of one’s weight perception on the number of calories chosen and on dieting, our study does have some limitations. Our results would be strengthened by using objective height and weight data. A chief limitation is that participants completed a hypothetical sandwich choice. If the way participants process food choice tasks in real life is different from the way they behave in a hypothetical task, the results in this study might not reflect what we would observe in real-life settings such as restaurants or at home. However, some features of food choice may counteract this hypothetical bias. Food choice tends to rely fairly heavily on habitual decision-making infrastructure [42]. Individuals process anticipated taste information more rapidly than they process objective health information about food [43]. Prompting individuals to consider taste when making food choices results in choices that are no different from choices in a no-prompt condition, but choices when prompted to consider health are healthier and brain activity shows marked differences [44]. These results suggest that people quickly and naturally incorporate taste attributes in their decision-making processes in a way that does not occur with health attributes.

Our findings suggest future research opportunities. For instance, all participants were observed making a sandwich choice. While there are many popular sandwich chains, competing fast-food restaurants offer items that may—on the whole—be less healthy, such as hamburgers or fried chicken. Variation in calories ordered might have been limited by the fact that all participants were limited to this choice environment. Providing a wider range of food options may result in greater differences in the nutritional attributes of individuals’ choices. Additionally, this study focuses only on habitual—i.e., reported dieting—and acute—that is, the number of calories ordered—weight control behaviors. Since a healthy diet is only half of the typical prescription for maintaining healthy body weight—the other being physical activity, it would be valuable to study simultaneous choices related to diet and exercise.

## Figures and Tables

**Table 1:** Sandwich ingredients and calories in food choice experiment.

Category	Item (Calories)
Meat/ protein	Bacon (254); salami (230); roast beef (207); roast turkey (180); ham (178); prosciutto (140); tofu (90)
Cheese	Cheddar cheese (115); Colby cheese (112); Swiss cheese (111); American cheese (104); provolone cheese (98); mozzarella cheese (85); light American cheese (36)
Spread/ dressing	Mayonnaise (188); Olive oil (119); Light mayonnaise (71); Italian dressing (35); Balsamic vinegar (14); Dijon mustard (10); Yellow mustard (6)
Bread	Croissant (406); Sourdough (319); Multigrain (265); Ciabatta (263); Bagel (250); Marble rye (233); Gluten-free (222)
Vegetables	Avocado (47); Red onion (11); Red pepper (8); Spinach (7); Tomato (5); Lettuce (4); Cucumber (3)

Source: Sandwich experiment.

**Table 2:** Demographic and diet-related information.

	Full Sample		Subsample: Normal Weight and Overweight	
Variables	Mean	Standard Deviation	Mean	Standard Deviation
Female (1 = yes)	0.51	0.50	0.48	0.50
Age (years)	36.91	11.12	36.46	11.20
Education (years)	14.80	1.71	14.88	1.68
Income ($1000s)	52.65	36.02	54.38	36.38
Dieting	0.240	0.427	0.211	0.408
Mean Calories Ordered	603.26	163.86	621.42	127.85
*N*	1044		712	

**Table 3:** Comparison between measured weight status and perceived weight status.

		Perceived Weight Status (%)			
Measured Weight Status (%)	Underweight (*n* = 33)	Normal Weight (*n* = 452)	Overweight (*n* = 398)	Obese (*n* = 138)	Total
Underweight (*n* = 26)	57.7	42.3	0.0	0.0	100.0
Normal weight (*n* = 418)	4.3	82.8	12.7	0.0	100.0
Overweight (*n* = 292)	0.0	29.1	69.2	1.7	100.0
Obese (*n* = 285)	0.0	3.5	46.3	50.2	100.0

Source: Data from the survey and food choice experiment.

**Table 4:** Relationship between weight status and dieting.

	Full Dataset		Subsample: Normal Weight and Overweight	
	Odds Ratio	95% Confidence Interval	Odds Ratio	95% Confidence Interval
(Intercept)	0.10	[0.06, 0.16]	0.12	[0.07, 0.22]
Overweight	2.57	[1.75, 3.78]	2.38	[1.61, 3.53]
Obese	3.37	[2.23, 5.11]	_	
Perception (Accurate)	1.74	[1.20, 2.55]	1.49	[0.88, 2.64]
Perception (Overestimate)	2.72	[1.31, 5.48]	2.35	[1.01, 5.38]
*N*	1021		710	
AIC	1084.7		722.7	

Source: Data from the survey and food choice experiment.

**Table 5:** Relationship between weight status, discrepancy between perceived and measured weight status, and calories ordered.

Coefficients	Full Sample	Subsample: Normal Weight and Overweight
(Intercept)	620.39 *** (14.94)	639.52 *** (18.32)
Overweight	3.07 (10.24)	−0.48 (10.37)
Obese	−13.27 (11.28)	_
Perception (Accurate)	−9.16 (10.60)	−32.09 * (14.37)
Perception (Overestimate)	−46.71 * (19.35)	−80.90 *** (22.07)
Adjusted R2	0.02	0.03
*N*	1021	710

Source: Data from the survey and food choice experiment. Note: We control for information conditions in both regressions. Significance codes: *** ≤ 0.001; * < 0.05.

## Data Availability

Data are available upon request from the corresponding author.

## Appendix A. Instructions for Sandwich Choice Exercise Conditions

### Appendix A.1. Control

Imagine you want to eat a sandwich, and you are given the following list of ingredients. Please select the ingredients that you want to add to your sandwich. You may choose only one ingredient per food category. Simply click on the box next to the ingredient to select an ingredient. The categories you will select from are:

1. Meat/Protein
2. Cheese
3. Spread/Dressing
4. Bread
5. Vegetables

### Appendix A.2. Calorie Info

Imagine you want to eat a sandwich, and you have the following list of ingredients available to build the sandwich. Please select the ingredients that you want to add to your sandwich. You may only choose one ingredient per food category. Simply click on the box next to the ingredient to select an ingredient. The number of calories that each ingredient will add to the sandwich is presented in parentheses behind the ingredient. The categories you will select from are:

1. Meat/Protein
2. Cheese
3. Spread/Dressing
4. Bread
5. Vegetables

### Appendix A.3. Min Ref

Imagine you want to eat a sandwich, and you have the following list of ingredients available to build the sandwich. Please select the ingredients that you want to add to your sandwich. You may only choose one ingredient per food category. Simply click on the box next to the ingredient to select an ingredient. Calorie information is presented in reference to the lowest calorie ingredient in each category. That ingredient is presented first, and the calorie information for all other ingredients in a category show the number of calories you will increase intake by if you select something other than the lowest calorie option. The categories you will select from are:

1. Meat/Protein
2. Cheese
3. Spread/Dressing
4. Bread
5. Vegetables

### Appendix A.4. Max Ref

Imagine you want to eat a sandwich, and you have the following list of ingredients available to build the sandwich. Please select the ingredients that you want to add to your sandwich. You may only choose one ingredient per food category. Simply click on the box next to the ingredient to select an ingredient. Calorie information is presented in reference to the highest calorie ingredient in each category. That ingredient is presented first, and the calorie information for all other ingredients in a category show the number of calories you will reduce intake by if you select something other than the highest calorie option. The categories you will select from are:

1. Meat/Protein
2. Cheese
3. Spread/Dressing
4. Bread
5. Vegetables

### Appendix A.5. Calorie Summation

Imagine you want to eat a sandwich, and you have the following list of ingredients available to build the sandwich. Please select the ingredients that you want to add to your sandwich. You may only choose one ingredient per food category. Simply click on the box next to the ingredient to select an ingredient. The number of calories that each ingredient will add to the sandwich is presented in parentheses behind the ingredient and the sum of the calories from the ingredients you have selected will be presented in a box on the left-hand side of the screen. The categories you will select from are:

1. Meat/Protein
2. Cheese
3. Spread/Dressing
4. Bread
5. Vegetables

## Appendix B

**Table A1:** Regression results including variable capturing the amount of time since the individual had last eaten.

Coefficients	Full Sample	Subsample: Normal Weight and Overweight
(Intercept)	620.24 *** (15.39)	636.95 *** (18.71)
Overweight	3.05 (10.25)	−0.96 (10.40)
Obese	−13.29 (11.30)	_
Perception (Accurate)	−9.18 (10.62)	−32.95 * (14.43)
Perception (Overestimate)	−46.72 * (19.36)	−81.56 *** (22.10)
Time since last eaten (hrs.)	0.04 (0.97)	0.82 (1.20)
Adjusted R2	0.02	0.03
N	1021	710

Source: Data from the survey and food choice experiment. Note: We control for information conditions in both regressions. Significance codes: *** ≤ 0.001; * < 0.05.

## References

[1] Fryar C.D., Carroll M.D., Afful J. (2020). Prevalence of Overweight, Obesity, and Extreme Obesity among Adults Aged 20 and Over: United States, 1960–1962 through 2017–2018.

[2] Hruby A., Manson J.E., Qi L., Malik V.S., Rimm E.B., Sun Q., Willett W.C., Hu F.B. (2016). Determinants and Consequences of Obesity. Am. J. Public Health.

[3] Kim D.D., Basu A. (2016). Estimating the Medical Care Costs of Obesity in the United States: Systematic Review, Meta-Analysis, and Empirical Analysis. Value Health.

[4] Wu Y.-K., Berry D.C. (2018). Impact of weight stigma on physiological and psychological health outcomes for overweight and obese adults: A systematic review. J. Adv. Nurs..

[5] Preston S.H., Vierboom Y.C., Stokes A. (2018). The role of obesity in exceptionally slow US mortality improvement. Proc. Natl. Acad. Sci. USA.

[6] Finley C.E., Barlow C.E., Greenway F.L., Rock C.L., Rolls B.J., Blair S.N. (2007). Retention rates and weight loss in a commercial weight loss program. Int. J. Obes..

[7] DellaVigna S., Malmendier U. (2006). Paying not to go to the gym. Am. Econ. Rev..

[8] Moroshko I., Brennan L., O’Brien P. (2011). Predictors of dropout in weight loss interventions: A systematic review of the literature. Obes. Rev..

[9] Elfhag K., Rössner S. (2005). Who succeeds in maintaining weight loss? A conceptual review of factors associated with weight loss maintenance and weight regain. Obes. Rev..

[10] Elfhag K., Rössner S. (2010). Initial weight loss is the best predictor for success in obesity treatment and sociodemographic liabilities increase risk for drop-out. Patient Educ. Couns..

[11] Kuk J.L., Ardern C.I., Church T.S., Hebert J.R., Sui X., Blair S.N. (2009). Ideal weight and weight satisfaction: Association with health practices. Am. J. Epidemiol..

[12] Sharma S., Wharton S., Forhan M., Kuk J.L. (2011). Influence of weight discrimination on weight loss goals and self-selected weight loss interventions. Clin. Obes..

[13] Wharton C.M., Adams T., Hampl J.S. (2008). Weight loss practices and body weight perceptions among US college students. J. Am. Coll. Health.

[14] Lemon S.C., Rosal M.C., Zapka J., Borg A., Andersen V. (2009). Contributions of weight perceptions to weight loss attempts: Differences by body mass index and gender. Body Image.

[15] Chen H.-Y., Lemon S.C., Pagoto S.L., Barton B.A., Lapane K.L., Goldberg R.J. (2014). Personal and parental weight misperception and self-reported attempted weight loss in US children and adolescents, National Health and Nutrition Examination Survey, 2007–2008 and 2009–2010. Prev. Chronic Dis..

[16] Yaemsiri S., Slining M.M., Agarwal S.K. (2011). Perceived weight status, overweight diagnosis, and weight control among US adults: The NHANES 2003–2008 Study. Int. J. Obes..

[17] Nair D., Hart A. (2018). Family Physicians’ Perspectives on Their Weight Loss Nutrition Counseling in a High Obesity Prevalence Area. J. Am. Board Fam. Med..

[18] Ward Z.J., Long M.W., Resch S.C., Gortmaker S.L., Cradock A.L., Giles C., Hsiao A., Wang Y.C. (2016). Redrawing the US Obesity Landscape: Bias-Corrected Estimates of State-Specific Adult Obesity Prevalence. PLoS ONE.

[19] Gorber S.C., Tremblay M., Moher D., Gorber B. (2007). A comparison of direct vs. self-report measures for assessing height, weight and body mass index: A systematic review. Obes. Rev..

[20] Block J.P., DeSalvo K.B., Fisher W.P. (2003). Are physicians equipped to address the obesity epidemic? knowledge and attitudes of internal medicine residents. Prev. Med..

[21] Brener N.D., Eaton D.K., Lowry R., McManus T. (2004). The association between weight perception and BMI among high school students. Obes. Res..

[22] Gustafson C.R., Zeballos E. (2020). The effect of presenting relative calorie information on calories ordered. Appetite.

[23] Gustafson C.R., Zeballos E. (2019). Cognitive aids and food choice: Real-time calorie counters reduce calories ordered and correct biases in calorie estimates. Appetite.

[24] Gustafson C.R., Zeballos E. (2018). The effect of ingredient-specific calorie information on calories ordered. Prev. Med. Rep..

[25] FoodData Central. https://ndb.nal.usda.gov/.

[26] R Core Team (2021). R: A Language and Environment for Statistical Computing.

[27] Turner M.M., Skubisz C., Pandya S.P., Silverman M., Austin L.L. (2014). Predicting visual attention to nutrition information on food products: The influence of motivation and ability. J. Health Commun..

[28] Naughton P., McCarthy S.N., McCarthy M.B. (2015). The creation of a healthy eating motivation score and its association with food choice and physical activity in a cross sectional sample of Irish adults. Int. J. Behav. Nutr. Phys. Act..

[29] Duncan D.T., Wolin K.Y., Scharoun-Lee M., Ding E.L., Warner E.T., Bennett G.G. (2011). Does perception equal reality? Weight misperception in relation to weight-related attitudes and behaviors among overweight and obese US adults. Int. J. Behav. Nutr. Phys. Act..

[30] Rancourt D., Thurston I.B., Sonneville K.R., Milliren C.E., Richmond T.K. (2017). Longitudinal impact of weight misperception and intent to change weight on body mass index of adolescents and young adults with overweight or obesity. Eat. Behav..

[31] Conner M., Martin E., Silverdale N., Grogan S. (1996). Dieting in adolescence: An application of the theory of planned behaviour. Br. J. Health Psychol..

[32] Diez-Sampedro A., Olenick M., Maltseva T., Flowers M. (2019). A Gluten-Free Diet, Not an Appropriate Choice without a Medical Diagnosis. J. Nutr. Metab..

[33] Burrows A., Cooper M. (2002). Possible risk factors in the development of eating disorders in overweight pre-adolescent girls. Int. J. Obes. Relat. Metab. Disord..

[34] Nicoli M.G., Junior R.D.R.L. (2011). Binge Eating Disorder and body image perception among university students. Eat. Behav..

[35] Lim H., Lee H.-J., Park S., Kim C.-I., Joh H.-K., Oh S.W. (2014). Weight misperception and its association with dieting methods and eating behaviors in South Korean adolescents. Nutr. Res. Pract..

[36] Woo J. (2014). A survey of overweight, body shape perception and eating attitude of Korean female university students. J. Exerc. Nutr. Biochem..

[37] Bhanji S., Khuwaja A.K., Siddiqui F., Azam I., Kazmi K. (2011). Underestimation of weight and its associated factors among overweight and obese adults in Pakistan: A cross sectional study. BMC Public Health.

[38] Arslain K., Gustafson C.R., Baishya P., Rose D.J. (2021). Determinants of gluten-free diet adoption among individuals without celiac disease or non-celiac gluten sensitivity. Appetite.

[39] Block J.P., Condon S.K., Kleinman K., Mullen J., Linakis S., Rifas-Shiman S., Gillman M.W. (2013). Consumers’ estimation of calorie content at fast food restaurants: Cross sectional observational study. BMJ.

[40] Moran A.J., Ramirez M., Block J.P. (2017). Consumer underestimation of sodium in fast food restaurant meals: Results from a cross-sectional observational study. Appetite.

[41] Jo J., Lusk J.L., Muller L., Ruffieux B. (2016). Value of parsimonious nutritional information in a framed field experiment. Food Policy.

[42] Rangel A. (2013). Regulation of dietary choice by the decision-making circuitry. Nat. Neurosci..

[43] Sullivan N., Hutcherson C., Harris A., Rangel A. (2015). Dietary self-control is related to the speed with which attributes of healthfulness and tastiness are processed. Psychol. Sci..

[44] Hare T.A., Malmaud J., Rangel A. (2011). Focusing attention on the health aspects of foods changes value signals in vmPFC and improves dietary choice. J. Neurosci..

